# Hypertrophy of the Tongue

**Published:** 1832

**Authors:** 


29. *Hypertrophy of the Tongue.* By Dr. Wells.—A girl, six years of age, was brought to Lexington for professional assistance, in May, 1829, with an enormous enlargement of the tongue.

The following are the dimensions and condition of the tongue at the time: length as it hung down over the chin from the superior incisors to its apex, 2 1-2 inches; breadth from one angle to the mouth of the other, a little more than two inches. The structure was more dense than natural; the upper surface smooth; the lower covered with the cicatrices of sores from the pressure of the teeth. The increase of size within the mouth had been less marked. She had formerly suffered much from inflammation and ulceration of the mucous membrane of the tongue which had been obviated by keeping it covered with cloth bags, but if these were omitted for a few days, the surface immediately became very sore. The front teeth had been displaced by the pressure of the tongue, and the anterior portion of the upper maxillary bone slightly curved upward, and of the lower jaw still more downward, so that while the back teeth were closed, the front teeth were an inch asunder.

This state of things was brought about by repeated attacks of glossitis.

The first proposition of Dr. Wells was to remove the protruding part of the tongue by one stroke, but the unwillingness of the patient and her friends to submit to this, induced him to propose the ligature.

The child having been freely purged and kept on a low diet for several days, a seton needle half an inch broad was carried through the tongue from below upward and obliquely backward so as to give the remaining portion of the tongue a pointed appearance. When the ligatures were firmly tied, the flow of blood which at first was considerable, ceased. Twenty hours afterward the strangulated portion was removed by the bistoury. Considerable irritation followed the application of the ligature, requiring antiphlogistic treatment, which soon ceased after the removal of the strangulated portion. The wound was dressed with lint wet with a solution of chloride of lime, and soon healed. In five or six weeks the patient had acquired perfect command of the lips and jaws, and could articulate the letters of the alphabet, and when Dr. Wells saw her a few months afterward, the jaws were restored to their natural shape, all the teeth coming in contact when the mouth was closed.

Dr. W. states that there is but one case of this kind on record in in the journals of this country, viz: one reported by Dr. Harris in a late No. of the American Journal. This case, however, differed in some important particulars, especially in the fact of the enlargement at the commencement not being of an intermittent character, and in its being accompanied with a change of structure, which was not the fact in the case of Dr. Wells.

